# Central obesity is associated with lower prevalence of sarcopenia in older women, but not in men: a cross-sectional study

**DOI:** 10.1186/s12877-022-03102-7

**Published:** 2022-05-09

**Authors:** Seongmin Choi, Jinmann Chon, Seung Ah Lee, Myung Chul Yoo, Yeocheon Yun, Sung Joon Chung, Minjung Kim, Eun Taek Lee, Min Kyu Choi, Chang Won Won, Yunsoo Soh

**Affiliations:** 1 Department of Physical Medicine and Rehabilitation, Kyung Hee University Medical Center, 23 Kyungheedae-ro, Dongdaemoon-gu, Seoul, 02447 Republic of Korea; 2 Department of Physical Medicine and Rehabilitation, Kyung Hee University Hospital at Gangdong, Seoul, Republic of Korea; 3 Department of Family Medicine, Kyung Hee University Medical Center, 23 Kyungheedae-ro, Dongdaemoon-gu, Seoul, 02447 Republic of Korea

**Keywords:** Aging, Sarcopenia, Obesity, Central obesity, Asian Working Group for Sarcopenia

## Abstract

**Background:**

Obesity is a chronic disease that causes various medical health problems, increases morbidity, and reduces the quality of life. Obesity (especially central obesity) in older adults is expected to act with the development of sarcopenia. However, the relationship between obesity, central obesity, and sarcopenia remains controversial. This study aimed to investigate the impact of obesity on sarcopenia.

**Methods:**

In this cross-sectional study, we used data from the Korean Frailty and Aging Cohort Study; 1,827 community-dwelling older adults (883 men and 944 women) aged 70–84 years were recruited. The Asian Working Group for Sarcopenia (AWGS) criteria were used to evaluate sarcopenia. Subjects with a low appendicular skeletal muscle mass index (ASMI; men: < 7.0 kg/m^2^, women: < 5.4 kg/m^2^) and either low handgrip strength (HGS; men: < 28 kg, women: < 18 kg) or low Short Physical Performance Battery (SPPB; ≤ 9) were diagnosed with sarcopenia. Obesity was defined as a body mass index (BMI) of ≥ 25 kg/m^2^, while central obesity was defined as WC measurements of ≥ 90 cm in men and ≥ 85 cm in women. Logistic regression analyses were performed to evaluate the impact of obesity and central obesity on sarcopenia and the parameters of sacropenia.

**Results:**

In both sexes, the obese group, defined based on the BMI, had a significantly low prevalence of low ASMI (odds ratio [OR] = 0.14, 95% confidence interval CI = 0.10–0.20 in men, OR = 0.17, 95% CI = 0.12–0.25 in women) and sarcopenia (OR = 0.28, 95% CI = 0.16–0.50 in men, OR = 0.17, 95% CI = 0.08–0.35 in women) in the multivariable logistic regression analysis. In women, the central obese group had a low prevalence of sarcopenia (OR = 0.46, 95% CI = 0.27–0.77) in the multivariable logistic regression analysis. Meanwhile, the obese group had a significantly higher prevalence of low SPPB in women (OR = 1.75, 95% CI = 1.18–2.59).

**Conclusions:**

Obesity may have a protective effect on low ASMI and sarcopenia, as defined by the AWGS criteria. Central obesity was associated with a low prevalence of sarcopenia in women only. However, obesity did not have a positive impact on functional parameters of sarcopenia including muscle strength and physical performance.

## Background

The prevalence of obesity in old age increases with socioeconomic development, a preference for convenient food, and reduced physical activity [1]. In Korea, the prevalence rates of obesity in older adults aged 70–79 years with a body mass index (BMI) of 25 kg/m^2^ or more increased from 27.4% in men and 38.7% in women in 2009 to 36.3% in men and 42.9% in women in 2018, showing a steady increase in both sexes [1]. Obesity is a chronic disease that causes various medical health problems, increases morbidity, and reduces the quality of life. Prevention and management of obesity are especially important in older adults. On the contrary, some studies have reported the beneficial effect of obesity on the lifespan of older adults [2, 3].

With aging, not only does body fat increase, but it is also distributed differently. The amount of intra-abdominal, intramuscular, and liver fat increases. Lean body mass decreases up to 3 kg per decade after 50 years (especially the muscle mass of the lower extremities), even without changes in body weight [4]. Waist circumference (WC), an indicator of central obesity, also known as abdominal obesity, is an alternative method to evaluate obesity in older adults. The diagnostic cutoff values for central obesity in South Korea are 90 cm for men and 85 cm or more for women, which are associated with a high risk for diabetes, hypertension, and dyslipidemia [1]. Central obesity also increases the risk of mortality due to diabetes and coronary artery disease, independent of BMI. The prevalence of central obesity in individuals aged 70–79 years increased from 19.0% in 2009 to 23.8% in 2018 in South Korea [1]. A decrease in muscle mass can lead to a reduction in muscle strength, physical performance, and sarcopenia [5]. Central obesity is also associated with sarcopenia by increasing fat accumulation in the skeletal muscle, which increases the level of inflammatory cytokines, such as tumor necrosis factor-alpha and interleukin-6, and inhibits muscle protein anabolism and increases insulin resistance [6–8]. However, a previous study showed that central obesity in older women was protective against developing sarcopenia [9].

Obesity (especially central obesity) in older adults is expected to occur with the development of sarcopenia [10]. However, the relationship between obesity, central obesity, and sarcopenia remains controversial. To the best of our knowledge, an association between obesity and the component parameters of sarcopenia has not yet been performed. Therefore, this study aimed to investigate the impact of obesity on the risk of developing sarcopenia, defined based on the 2019 Asian Working Group for Sarcopenia (AWGS) criteria in community-dwelling Korean older adults using data from the Korean Frailty and Aging Cohort Study (KFACS).

## Methods

### Data and study population

**Fig. 1:**
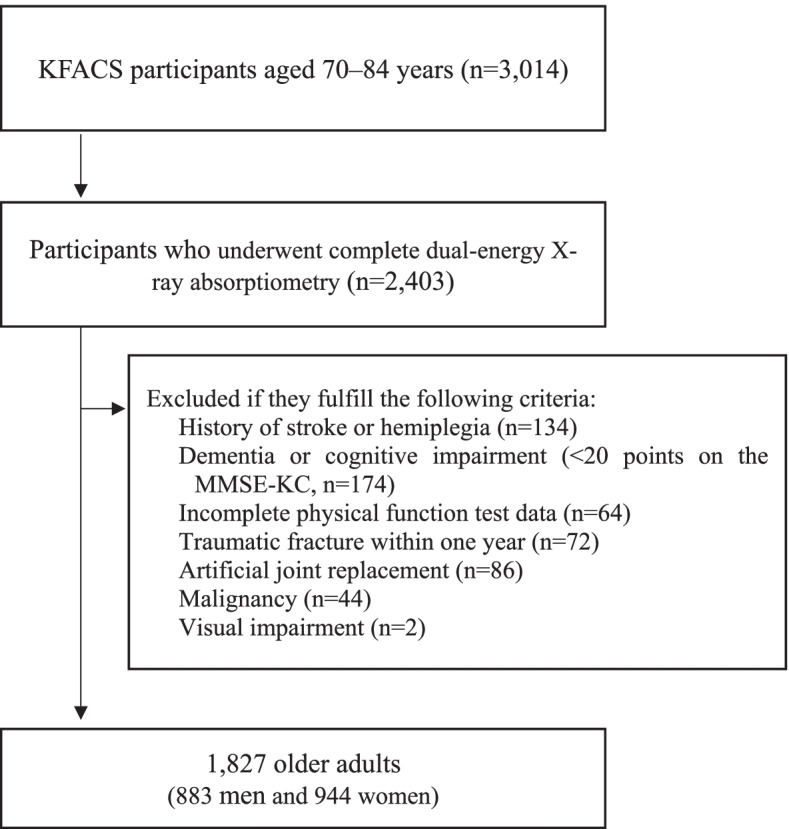
Flow chart of the participant recruitment process. KFACS, Korean Frailty and Aging Cohort Study; MMSE-KC, Mini-Mental Status Examination in the Korean version of the CERAD assessment packet

Data from the 2016 to 2017 KFACS was used in this cross-sectional study. The KFACS, a nationwide multicenter study, was conducted in community-dwelling older adults aged 70–84 years from eight hospitals and two public health centers across South Korea. Among the 3,014 participants, 2,403 who completed dual-energy X-ray absorptiometry (DXA) were included. Participants with incomplete data on weakness due to stroke, a deformity or motor deficit in the extremities, dementia or cognitive impairment, blindness, malignancy, a traumatic fracture within 1 year, history of artificial joint replacement, and inability to complete the short physical performance battery (SPPB) test were excluded. Overall, 1,827 participants (883 men and 944 women) were included in the final analysis (Fig. 1). The demographic data and medical history, including age, sex, marital status, income per month, location of residence (rural or city), BMI, WC, and chronic comorbidities, smoking, and alcohol status, were obtained from each participants. Participants who smoked more than one cigarette per week and drank alcohol at least once a week were defined as smokers and drinkers, respectively.

The KFACS protocol was approved by the Institutional Review Board (IRB) of the Clinical Research Ethics Committee of Kyung Hee University Medical Center (IRB number: 2022–01-041), and all participants provided written informed consent.

### Sarcopenia

The prevalence of sarcopenia and the parameters of sarcopenia, including muscle mass, muscle strength, and physical performance, were used as outcomes. Sarcopenia was defined according to the AWGS 2019 diagnostic criteria [11]. Subjects with a low appendicular skeletal muscle mass (ASM) and either low muscle strength or low physical performance were diagnosed with sarcopenia. Subjects with a low ASM, low muscle strength, and low physical performance (SPPB ≤ 9) were further defined as having severe sarcopenia.

1. Muscle mass: DXA was used to measure the ASM, and ASM index (ASM/height^2^, ASMI) was calculated to compare the muscle mass at different heights (cutoff values men: < 7.0 kg/m^2^, women: < 5.4 kg/m^2^).
2. Muscle strength: Handgrip strength (HGS) was measured using a hand dynamometer (Jamar, Bolingbrook, IL, USA). HGS measurement was performed twice on both sides, with the elbow flexed to 90° in a sitting position, and the maximum value was obtained (cutoff values, men: < 28 kg, women: < 18 kg) [12].
3. Physical performance The Short Physical Performance Battery (SPPB) was used to assess physical performance. The SPPB is a well-established test that evaluates the function of the lower extremities in older adults. This test includes the following items: standing balance, 4-m gait speed, and five times sit to stand test. Each item was scored 4 points, with a higher score indicating better lower extremity physical performance. According to the AWGS diagnostic criteria, a score of ≤ 9 points was defined as low physical performance [13].

### Obesity

Obesity was classified according to the BMI. BMI was calculated as body weight (kg) divided by height in meters squared (m^2^). The cutoff values for defining obesity are lower for Asians than that for non-Asians. Because individuals with a BMI of ≥ 25 kg/m^2^ have a significant risk for obesity-related disease, obesity was defined as a BMI of ≥ 25 kg/m^2^ according to the Asia–Pacific criteria of the World Health Organization guidelines [14]. Central obesity was defined as WC measurements of ≥ 90 cm in men and ≥ 85 cm in women, according to the Korean Society for the Study of Obesity (KSSO) definition [15]. WC was measured horizontally midway between the superior iliac crest and the lower margin of the 12^th^ rib.

### Statistical analysis

Continuous variables were compared using the t-test, while categorical variables were compared using Pearson’s chi-square test. Continuous variables were expressed as means ± standard deviations, while categorical variables were expressed as numbers and ratios (%). Unadjusted and fully adjusted analyses were performed using logistic regression models, and the odds ratios (ORs) and 95% confidence intervals (CIs) were calculated. Each model was adjusted for potential confounding variables, such as age, hypertension, dyslipidemia, osteoarthritis, osteoporosis, diabetes mellitus, smoking history, alcohol history, location of residence, family group, number of medications, and score on Mini-Mental Status Examination in the Korean version (MMSE-KC). All statistical analyses were performed using Statistical Package for Social Sciences (version 25.0; SPSS Inc., Chicago, Illinois, USA); a *P*-value of < 0.05 was considered significant.

## Results

The baseline characteristics of the patients are presented in Table 1. Among the 1,827 patients, 883 (48%) were men and 944 (52%) were women. The prevalence of obesity, defined by BMI, was significantly higher in women than in men (35.3% vs. 45.0%, *P* < 0.01). The prevalence of central obesity in women was significantly higher than that in men (64.5% vs. 38.1%, *P* < 0.01). Other socioeconomic characteristics, including marital status, monthly income, location of residence, smoking habits, and chronic comorbidities, were significantly different between the groups.

**Table 1:** Baseline characteristics of the participants according to sex

		**Men**	**Women**	**Total**	***P*-value**
		**(*n* = 883)**	**(*n* = 944)**	**(*n* = 1,827)**	
Age (years)		76.5 ± 3.7	75.7 ± 3.7	76.1 ± 3.8	0.21
BMI (kg/cm^2^)		24.0 ± 2.8	24.8 ± 2.9	24.5 ± 2.9	0.47
Waist circumference (cm)		87.6 ± 8.2	87.9 ± 8.3	87.8 ± 8.3	0.41
Height (cm)		165.1 ± 5.4	152.2 ± 5.1	158.5 ± 8.4	< 0.01*
Weight (kg)		65.7 ± 8.7	57.6 ± 7.7	61.6 ± 9.2	< 0.01*
Obesity by BMI					
Normal (BMI < 25)		571 (64.7)	519 (55.0)	1090 (59.7)	< 0.01*
Obese (30 > BMI ≥ 25)		290 (32.8)	381 (40.4)	671 (36.7)	
Severe obese (BMI ≥ 30)		22 (2.5)	44 (4.7)	66 (3.6)	
Central obesity by WC					
No (men < 90, women < 85)		547 (61.9)	335 (35.5)	882 (48.3)	< 0.01*
Yes (men ≥ 90, women ≥ 85)		336 (38.1)	609 (64.5)	945 (51.7)	
Marriage (%)	Married	816 (92.4)	626 (66.3)	1442 (78.9)	< 0.01*
	Not married	67 (7.6)	318 (33.7)	385 (21.1)	
Income per month	More than 3	181 (20.5)	177 (18.8)	358 (19.6)	< 0.01*
(Korean million won, %)	1–3	448 (50.7)	373 (39.5)	821 (44.9)	
	Less than 1	254 (28.8)	394 (41.7)	648 (35.5)	
Location of residence (%)	Urban	690 (78.1)	806 (85.4)	1496 (81.9)	< 0.01*
	Rural	193 (21.9)	138 (14.6)	331 (18.1)	
Current smoker (%)		573 (67.9)	18 (1.9)	591 (32.3)	< 0.01*
Alcohol use (%)		539 (60.9)	536 (56.8)	1074 (58.8)	0.72
Hypertension (%)		459 (52.0)	552 (58.5)	1011 (55.3)	0.01*
Dyslipidemia (%)		222 (25.1)	402 (42.6)	624 (34.2)	< 0.01*
Diabetes mellitus (%)		214 (24.2)	175 (18.5)	389 (21.3)	< 0.01*
Depression (%)		15 (1.7)	34 (3.6)	49 (2.7)	0.01*
Knee OA (%)		90 (10.2)	296 (31.4)	386 (21.1)	< 0.01*
Osteoporosis (%)		23 (2.6)	227 (24.0)	250 (13.7)	< 0.01*
MMSE-KC		26.4 ± 2.7	25.6 ± 3.1	26.0 ± 3.0	< 0.01*

*Abbreviations*: *BMI* Body mass index, *OA* Osteoarthritis, *MMSE-KC* Mini-Mental Status Examination-Korean version, *WC* Waist circumference

A comparison of the obese and non-obese groups according to sarcopenia status and its component parameters, including HGS, ASMI, and SPPB, is shown in Table 2. In men, the obese group defined by BMI had significantly higher HGS and ASMI and a lower prevalence of sarcopenia than the non-obese group. In women, the obese group defined by BMI had significantly higher ASMI, lower SPPB, and lower prevalence of sarcopenia than the non-obese group. In addition, the central obese group in women had higher ASMI and lower prevalence of sarcopenia than the non-obese group.

**Table 2:** Sarcopenia parameters of the obese and non-obese participants according to sex

Characteristic	Men			Women		
	Obese^a^ (*n* = 312)	Non-obese (*n* = 571)	*P*-value	Obese^a^ (*n* = 425)	Non-obese (*n* = 519)	*P*-value
HGS, kg (mean ± SD)	33.76 ± 5.35	33.00 ± 5.14	0.03*	22.13 ± 3.47	21.78 ± 3.48	0.12
ASMI, kg/m^2^ (mean ± SD)	7.62 ± 0.72	6.82 ± 0.72	< 0.01*	6.18 ± 0.65	5.60 ± 0.64	< 0.01*
SPPB (mean ± SD)	11.30 ± 1.10	11.25 ± 1.07	0.43	10.61 ± 1.53	10.93 ± 1.38	< 0.01*
Sarcopenia^c^ (n, %)	18 (5.8)	94 (16.5)	< 0.01*	10 (2.4)	57 (11.0)	< 0.01*
Severe sarcopenia^d^ (n, %)	3 (1.0)	11 (1.9)	0.270	3 (0.7)	11 (2.1)	0.07
	Central obesity^b^ (*n* = 336)	Non-obese (*n* = 547)	*P* value	Central obesity^b^ (*n* = 609)	Non-obese (*n* = 335)	*P* value
HGS, kg (mean ± SD)	33.40 ± 5.42	33.2 ± 5.1	0.60	21.97 ± 3.36	21.89 ± 3.69	0.73
ASMI, kg/m^2^ (mean ± SD)	7.09 ± 0.81	7.1 ± 0.8	0.52	5.89 ± 0.72	5.79 ± 0.68	0.04*
SPPB (mean ± SD)	11.17 ± 1.10	11.3 ± 1.1	0.27	10.83 ± 1.47	10.71 ± 1.43	0.25
Sarcopenia^c^ (n, %)	43 (12.8)	69 (12.6)	0.94	33 (5.4)	34 (10.1)	0.01*
Severe sarcopenia^d^ (n, %)	6 (1.8)	8 (1.5)	0.71	6 (1.0)	8 (2.4)	0.09
	Obese by both of BMI and WC (*n* = 115)	Non-obese (*n* = 768)	*P* value	Obese by both of BMI and WC (*n* = 282)	Non-obese (*n* = 662)	*P* value
HGS, kg (mean ± SD)	34.57 ± 5.40	33.1 ± 5.2	< 0.01*	22.01 ± 3.45	21.91 ± 3.50	0.67
ASMI, kg/m^2^ (mean ± SD)	7.67 ± 0.71	7.0 ± 0.8	< 0.01*	6.23 ± 0.64	5.70 ± 0.67	< 0.01*
SPPB (mean ± SD)	11.27 ± 1.09	11.3 ± 1.1	0.97	10.56 ± 1.61	10.88 ± 1.38	< 0.01*
Sarcopenia^c^ (n, %)	2 (1.7)	110 (14.3)	< 0.01*	4 (1.4)	63 (9.5)	< 0.01*
Severe sarcopenia^d^ (n, %)	1 (0.9)	14 (1.8)	0.51	1 (0.4)	13 (2.0)	0.06

*Abbreviations*: *BMI* Body mass index, *WC* Waist circumference, *HGS* Handgrip strength, *ASMI* Appendicular skeletal muscle mass index, *SPPB* Short physical performance battery

^a^BMI ≥ 25 kg/m^2^

^b^WC ≥ 90 cm for men and ≥ 85 cm for women

^c^Sarcopenia: low ASMI (< 7.0 kg/m^2^ for men and < 5.4 kg/m^2^ for women) and either a low HGS (< 28 kg for men and < 18 kg for women), or low physical performance (SPPB score ≤ 9 for both sexes)

^d^Severe sarcopenia: low ASMI with low HGS and low physical performance

^*^P < 0.05

Table 3 shows the results of the logistic regression analysis of obesity defined as a BMI ≥ 25 predicting sarcopenia and its parameters according to gender. The fully adjusted model showed significantly lower prevalence of low ASMI (odds ratio [OR] = 0.14, 95% confidence interval [CI] = 0.10–0.20 in men; OR = 0.17, 95% CI = 0.12–0.25 in women) and sarcopenia (OR = 0.28, 95% CI = 0.16–0.50 in men; OR = 0.17, 95% CI = 0.08–0.35 in women) in the obese group compared with that in the non-obese group.

**Table 3:** Logistic regression analysis of obesity defined by BMI predicting sarcopenia and its parameters according to sex^a^

Response variables	Unadjusted model		Fully adjusted model	
	Men	Women	Men	Women
	OR (95% CI)	OR (95% CI)	OR (95% CI)	OR (95% CI)
Low HGS^b^	0.90 (0.70–1.59)	0.71 (0.47–1.09)	0.84 (0.55–1.29)	0.70 (0.45–1.11)
Low ASMI^b^	0.16 (0.11–0.21)**	0.17 (0.12–0.25)**	0.14 (0.10–0.20)**	0.17 (0.12–0.25)**
Low SPPB^b^	1.05 (0.62–1.77)	1.39 (0.99–1.96)	1.11 (0.62–1.98)	1.40 (0.96–2.05)
Sarcopenia^c^	0.31 (0.18–0.53)**	0.20 (0.10–0.39)**	0.28 (0.16–0.50)**	0.17 (0.08–0.35)**
Severe sarcopenia^d^	0.49 (0.14–1.79)	0.33 (0.09–1.18)	0.51 (0.13–2.08)	0.25 (0.06–1.06)

*Abbreviations*: *OR* Odds ratio, *CI* Confidence interval, *HGS* Handgrip strength, *ASMI* Appendicular skeletal muscle mass index, *SPPB* Short physical performance battery

The fully adjusted model was adjusted for age, hypertension, dyslipidemia, osteoarthritis, osteoporosis, diabetes mellitus, smoking history, alcohol history, location of residence, family group, number of medications, and MMSE-KC score

^a^BMI ≥ 25 kg/m^2^

^b^Low HGS, < 28 kg for men and < 18 kg for women; low ASMI, < 7.0 kg/m^2^ for men and < 5.4 kg/m^2^ for women; low SPPB, score ≤ 9 for both sexes

^c^Sarcopenia: low ASMI (< 7.0 kg/m^2^ for men and < 5.4 kg/m^2^ for women) and either a low HGS (< 28 kg for men and < 18 kg for women), or low physical performance (SPPB score ≤ 9 for both sexes)

^d^Severe sarcopenia: low ASMI with low HGS and low physical performance

^*^P < 0.05

^**^P < 0.01

Table 4 shows the results of the logistic regression analysis of central obesity predicting sarcopenia and its parameters according to sex. In women, the central obese group had a significantly lower prevalence of sarcopenia in the unadjusted model (OR = 0.51, 95% CI = 0.31–0.84) and fully adjusted model (OR = 0.46, 95% CI = 0.27–0.77). In men, the prevalence of sarcopenia was not significantly lower in the central obese group in the unadjusted (OR = 1.02, 95% CI = 0.68–1.53) and fully adjusted models (OR = 1.04, 95% CI = 0.68–1.60).

**Table 4:** Logistic regression analysis of central obesity predicting sarcopenia and its parameters according to sex^a^

Characteristics	Unadjusted model		Fully adjusted model	
	Men	Women	Men	Women
	OR (95% CI)	OR (95% CI)	OR (95% CI)	OR (95% CI)
Low HGS^b^	0.95 (0.65–1.39)	0.75 (0.49–1.14)	0.98 (0.66–1.46)	0.69 (0.45–1.08)
Low ASMI^b^	1.02 (0.78–1.34)	0.85 (0.63–1.15)	1.03 (0.78–1.36)	0.82 (0.60–1.13)
Low SPPB^b^	1.49 (0.90–2.46)	0.94 (0.66–1.34)	1.64 (0.95–2.82)	0.95 (0.65–1.40)
Sarcopenia^c^	1.02 (0.68–1.53)	0.51 (0.31–0.84)**	1.04 (0.68–1.60)	0.46 (0.27–0.77)**
Severe sarcopenia^d^	1.23 (0.42–3.56)	0.41 (0.14–1.18)	1.19 (0.38–3.66)	0.35 (0.10–1.10)

*Abbreviations*: *OR* Odds ratio, *CI* Confidence interval, *HGS* Handgrip strength, *ASMI* Appendicular skeletal muscle mass index, *SPPB* Short physical performance battery

The fully adjusted model was adjusted for age, hypertension, dyslipidemia, osteoarthritis, osteoporosis, diabetes mellitus, smoking history, alcohol history, location of residence, family group, number of medications, and MMSE-KC score

^a^Waist circumference ≥ 90 cm for men and ≥ 85 cm for women

^b^Low HGS, < 28 kg for men and < 18 kg for women; low ASMI, < 7.0 kg/m^2^ for men and < 5.4 kg/m^2^ for women; low SPPB, score ≤ 9 for both sexes

^c^Sarcopenia: low ASMI (< 7.0 kg/m^2^ for men and < 5.4 kg/m^2^ for women) and either a low HGS (< 28 kg for men and < 18 kg for women), or low physical performance (SPPB score ≤ 9 for both sexes)

^d^Severe sarcopenia: low ASMI with low HGS and low physical performance

^*^P < 0.05

^**^P < 0.01

Results of the logistic regression analysis of obesity that met the criteria of both BMI and WC predicting sarcopenia and its parameters according to sex are shown in Table 5. In the fully adjusted model, both men and women with high BMI and central obesity had a significantly lower prevalence of low ASMI (OR = 0.21, 95% CI = 0.12–0.35 in men, OR = 0.18, 95% CI = 0.11–0.28 in women) and sarcopenia (OR = 0.10, 95% CI = 0.02–0.42 in men, OR = 0.12, 95% CI = 0.04–0.33 in women); in women, the obese group had a significantly higher prevalence of low SPPB (≤ 9) in the fully adjusted model (OR = 1.75, 95% CI = 1.18–2.59).

**Table 5:** Logistic regression analysis of obesity defined by both of BMI and waist circumference for sarcopenia and its parameters^a^

Characteristics	Unadjusted model		Fully adjusted model	
	Men	Women	Men	Women
	OR (95% CI)	OR (95% CI)	OR (95% CI)	OR (95% CI)
Low HGS^b^	0.74 (0.41–1.34)	0.84 (0.53–1.34)	0.70 (0.37–1.32)	0.83 (0.51–1.36)
Low ASMI^b^	0.20 (0.12–0.34)**	0.18 (0.12–0.29)**	0.21 (0.12–0.35)**	0.18 (0.11–0.28)**
Low SPPB^b^	1.06 (0.51–2.20)	1.66 (1.17–2.37)**	1.15 (0.52–2.56)	1.75 (1.18–2.59)**
Sarcopenia^c^	0.11 (0.03–0.44)**	0.14 (0.05–0.38)**	0.10 (0.02–0.42)**	0.12 (0.04–0.33)**
Severe sarcopenia^d^	0.51 (0.07–3.93)	0.18 (0.02–1.37)	0.53 (0.06–4.45)	0.14 (0.02–1.22)

*Abbreviations*: *OR* Odds ratio, *CI* Confidence interval, *HGS* Handgrip strength, *ASMI* Appendicular skeletal muscle mass index, *SPPB* Short physical performance battery

The fully adjusted model was adjusted for age, hypertension, dyslipidemia, osteoarthritis, osteoporosis, diabetes mellitus, smoking history, alcohol history, location of residence, family group, number of medications, and MMSE-KC score

^a^BMI ≥ 25 kg/m^2^; waist circumference ≥ 90 cm for men and ≥ 85 cm for women

^b^Low HGS, < 28 kg for men and < 18 kg for women; low ASMI, < 7.0 kg/m^2^ for men and < 5.4 kg/m^2^ for women; low SPPB, score ≤ 9 for both sexes

^c^Sarcopenia: low ASMI (< 7.0 kg/m^2^ for men and < 5.4 kg/m^2^ for women) and either a low HGS (< 28 kg for men and < 18 kg for women), or low physical performance (SPPB score ≤ 9 for both sexes)

^d^Severe sarcopenia: low ASMI with low HGS and low physical performance

^*^P < 0.05

^**^P < 0.01

## Discussion

This study investigated the associations between obesity according to the BMI criteria, central obesity by WC, and sarcopenia according to sex. In our study, a high BMI was associated with a low prevalence of low ASMI and sarcopenia, defined based on the AWGS criteria. Central obesity was associated with a low prevalence of sarcopenia in women. However, obesity was not found to be positively associated with muscle strength or physical performance. These results are consistent with those of previous studies showing that low BMI is positively associated with sarcopenia and that obesity has a positive effect on sarcopenia [16–18].

In both sexes, obesity, defined as high BMI, was associated with a low prevalence of low muscle mass and sarcopenia. The association between BMI and sarcopenia has been investigated in a few studies. According to previous studies, low BMI was positively related to sarcopenia, and obesity determined by BMI had a protective effect against sarcopenia. Senior et al. studied the risk factors of sarcopenia among adults living in nursing homes and found that low BMI was a predictive factor for sarcopenia [16]. Sato et al. conducted a cross-sectional study of older adults to investigate the risk factors for sarcopenia and showed that overweight defined by a BMI of ≥ 25 kg/m^2^ had a protective effect against sarcopenia (OR = 0.95, 95% CI = 0.00–0.06) [17]. In addition, Yu et al. examined the incidence of sarcopenia and its associated factors over 4 years and revealed that low BMI was associated with the development of sarcopenia during the 4-year follow-up (OR = 0.66, 95% CI = 0.62–0.70) [18].

The relationship between high BMI and ASMI can be explained by the following hypothesis. First, since BMI is calculated by dividing the body mass by the square of the height, individuals with high BMI have high body mass. High body mass in obese individuals may have a chronic overload stimulus on antigravity muscles helping maintain an upright, balanced position (e.g., quadriceps, calf), which increases the muscle size and strength [19, 20]. A few previous studies have revealed that a high BMI is significantly associated with stronger antigravity muscles and increased lower-limb skeletal muscle volume as measured by CT. However, handgrip strength was not significantly related to obesity [19, 20]. Second, since BMI cannot be used to distinguish lean mass from body fat mass, individuals with a high BMI can have a high lean mass. A relatively poor correlation between the percentage of body fat mass and BMI has been found in a few studies, and a previous study revealed that BMI correlated better with lean body mass than fat mass [21].

By contrast, another study showed an interaction between adipocytes and myocytes [22]. Increased leptin produced by adipocytes contributes to ectopic fat deposition in the muscles, leading to loss of muscle quality [23, 24]. The adipose tissue of an obese person has high levels of tumor necrosis factor-alpha, which promotes the production and secretion of several pro-inflammatory cytokines. Based on these pathways, fat gain and muscle loss seem to be linked to each other, while the adipocytes seem to have an interaction with myocytes [25]. Surprisingly, in this study, central obesity was associated with a low prevalence of sarcopenia only in women. In a previous study, Chen et al. reported that central obesity is associated with a lower risk of muscle mass loss in menopausal women [9]. Abdominal fat is thought to store high concentrations of sex hormones and has positive effects on skeletal muscle mass [26, 27]. Healthy adipocytes secrete adiponectin, an anti-inflammatory and insulin sensitizer, which is positively related to muscle mass [28]. This supports the hypothesis that our results are only applicable to women.

Other sarcopenia parameters, including HGS (representing muscle strength), SPPB (representing physical performance), and prevalence of severe sarcopenia, were not significantly associated with high BMI.

Additionally, in the obese group, defined by both BMI and WC, the results were not significantly different from those of obesity defined by high BMI, except for physical performance. The prevalence of low SPPB in the obese group was significantly higher than that in the non-obese group only in women. This finding indicates that obese women may have a higher probability of exhibiting lower physical performance, which is thought to be due to differences in body composition between the sexes. Since women have higher body fat and lower muscle mass, balance stability in this group may be more strongly affected by alterations in body fat composition than that in their men counterparts [29, 30]. In a previous study, Waters et al. found that poor balance was strongly associated with fat mass in women, whereas poor balance was associated with muscle mass in men [29].

In summary, high BMI was associated with a protective impact on low ASMI and sarcopenia defined based on the AWGS criteria, which may be due to the high ASMI in the obese group. Central obesity also had a protective impact against sarcopenia in women alone, which may be due to the effects of sex hormones and adiponectin. However, obesity was not positively associated with muscle strength or physical performance. Obesity and central obesity appear to have a protective effect against sarcopenia, as defined based on the AWGS criteria. However, since obesity does not have a positive impact on the functional parameters of sarcopenia, further studies are needed to investigate the association of clinical outcomes between obesity and sarcopenia.

This study has several limitations. First, this was a cross-sectional study. However, the results of this large cohort study with 1,827 participants are meaningful, as it is the first study to examine the association between obesity and sarcopenia and the component parameters of sarcopenia. Second, the association between body fat indices measured using dual-energy DXA and sarcopenia was not included in this study. Further prospective studies are required to confirm these findings. Third, in this study, the number of patients with severe obesity was relatively small (3.6% of the total participants); therefore, our results might not be properly reflected in the case of severe obesity. Lastly, this study might not apply to other races because it was conducted on a single race, the East Asian Korean population. Because body composition also differs between races, studies in other races are also warranted [31].

## Conclusion

This was the first large cross-sectional cohort study to investigate the association between obesity and the component parameters of sarcopenia. In this study, we found that a obesity may have a protective effect against low ASMI which is one of parameter of sarcopenia and sarcopenia defined based on the AWGS criteria. Central obesity was associated with a low prevalence of sarcopenia in women. On the contrary, obesity did not have a positive impact on muscle strength and physical performance.

## Data Availability

All cohort data that support the findings of this study are available from the KFACS and are open to all researchers on reasonable request. All published articles and news articles using the KFACS database, data provision manuals, and contact information are available on the KFACS website (http://www.kfacs.kr).

## Abbreviations

KFACS: Korean Frailty and Aging Cohort study
AWGS: Asian Working Group for Sarcopenia
SPPB: Short Physical Performance Battery
HGS: Handgrip strength
ASMI: Appendicular skeletal muscle mass index
BMI: Body mass index
EWGSOP: European Working Group on Sarcopenia in Older People
OR: Odds ratio
CI: Confidence interval
